# Influence of Cooking Conditions on Nutritional Properties and Sensory Characteristics Interpreted by E-Senses: Case-Study on Selected Vegetables

**DOI:** 10.3390/foods9050607

**Published:** 2020-05-09

**Authors:** Susanna Buratti, Carola Cappa, Simona Benedetti, Gabriella Giovanelli

**Affiliations:** Dipartimento di Scienze per gli Alimenti, la Nutrizione e l’Ambiente, Università degli Studi di Milano, Via G. Celoria, 2-20133 Milano, Italy; susanna.buratti@unimi.it (S.B.); simona.benedetti@unimi.it (S.B.); gabriella.giovanelli@unimi.it (G.G.)

**Keywords:** cauliflower, carrot, sweet potato, cooking method, antioxidant capacity, e-nose, e-tongue, e-eye, texture

## Abstract

This study investigates the effects of three cooking methods (boiling, steaming and microwaving) on the nutritional and physical properties of cauliflowers, carrots and sweet potatoes; e-senses were applied to interpret the sensory characteristics according to physico-chemical aspects. The nutritional quality was evaluated by determining the concentrations of key components and the antioxidant activity; e-sense data, combined with texture parameters, were processed by a principal component analysis. The cooking method and time significantly influenced the quality of the three products. Boiling, which detrimentally affected ascorbic acid, total phenolic concentration and antioxidant activity, enhanced carotene accessibility. Steaming produced losses in ascorbic acid, increasing total phenolics and carotenoids. Microwaving resulted in minor changes in ascorbic acid concentrations, preserved carotenoids and increased total phenolics. The nutritional quality was better preserved or enhanced using shorter cooking times. The elaboration of the data collected by the e-senses showed a clear evolution according to the cooking method and time. The results helped to determine the cooking method that best preserves the nutritional properties of the vegetables, highlighting the applicability of rapid instrumental methods to interpret the evolution of sensory characteristics.

## 1. Introduction

Vegetables are rich in phytochemicals and vitamins that are associated with a reduced risk of cardiovascular diseases, cancer and diabetes and thus contribute to a healthy and well-balanced diet. However, in order to improve the sensory characteristics and inactivate the antinutritional components (i.e., trypsin inhibitors), most vegetables (e.g., cauliflowers, beans, potatoes, etc.) need to be cooked before consumption [1,2]. Consequently, there is a growing interest in the effect of cooking on the nutritional and sensory quality of vegetables. The most popular cooking method is boiling but depending on the vegetable and consumer preferences, baking, roasting, steaming and microwaving are also commonly applied.

It is well known that the cooking process induces changes in the chemical composition of vegetables, influencing the concentration and bioavailability of bioactive compounds such as total phenolics and antioxidants [3]. The effects of cooking on several vegetables have been studied by various researchers, using different cooking techniques. However, it is difficult to come to unique conclusions about the advantages/disadvantages of a particular cooking method when the nutritional quality of vegetables is concerned. A study on the in vitro bioaccessibility of β-carotene from heat-processed orange-fleshed sweet potatoes reported that cooking (except microwaving) enhanced the release of carotenoids from the vegetable matrix by softening and degrading the cell walls [4]; the same results were obtained for cooked carrots and spinach [5]. Guillén et al. [6] reported that microwaving, stir-frying and boiling peppers (*Capsicum annum* L.) caused a marked difference in the radical scavenging activity, total phenol and ascorbic acid content of the cooked product, and that during boiling there was a leaching of antioxidant compounds from the peppers into the cooking water. The same authors reported a reduction in the carotenoid content in cooked peppers. Conversely, an enhancement of carotenoids bioavailability in cooked carrots and spinach, attributed to the ease of chemical extraction after cooking, was evidenced [5]. In general, many studies showed that all cooking methods improve some while compromising other nutritional and technological aspects of food (e.g., the leaching of soluble vitamins), suggesting that methods not requiring water immersion help to preserve the nutritional value of the product [7,8]. Another study, which focused on the color, pigments, total phenolic content and antioxidant activity in artichokes, green beans, broccoli and carrots cooked under different conditions, indicated that sous-vide cooking preserved chlorophyll, carotenoids, phenolic content and antioxidant activity to a greater extent than boiling and retained color better [9]. Unfortunately, sous-vide cooking is not easily usable as it requires vacuum packaging of the product before cooking. A great variability in the data concerning changes in vegetable nutrients after cooking has been reported in the literature [10] and it has been explained by the differences in vegetables’ materials, cooking parameters, extraction and the analysis procedure.

All the modifications induced by cooking impact on the physical and sensory properties of the cooked product; although these aspects play a crucial role in consumer acceptance and in the perceived quality of cooked vegetables, few studies have investigated these characteristics and little information is available on the relationship between nutritional quality and sensory properties. Cooking methods also differently affect product texture [7]. Some authors have studied the effect of cooking methods on the sensory attributes of selected vegetables evaluated by human panels [11,12,13,14], however, e-sense techniques have never been applied in these studies. E-senses have been developed to electronically reproduce responses similar to those of human senses. These include an electronic nose (e-nose), an electronic tongue (e-tongue) and an electronic eye (e-eye), which are widely applied in food analysis for quality assessment and process monitoring [15]. Several attempts have been made to combine electronic senses (e-senses) for food analysis and it has been demonstrated that their simultaneous utilization can give an overall picture of the sensorial quality of specific products [16].

The purpose of this study was to investigate the effects of different cooking methods (i.e., boiling, steaming and microwaving) on selected vegetables, in order to (i) define the best cooking method and time with regard to the nutritional properties of the products and (ii) show and interpret the changes in sensory properties, as evaluated by e-senses, according to physico-chemical characteristics. The cooking methods were chosen considering that boiling is the staple method, steaming is the most preferred technique to prevent the loss of water-soluble compounds and is appreciated by consumers [7,8] and microwaving is increasingly used thanks to its rapidity and ease of use. With respect to previously published studies, increasing cooking times were investigated for each cooking method, allowing us to compare the resultant changes. Three kinds of vegetables were examined: cauliflowers and carrots were selected as good sources of bioactive compounds that are widely consumed all over the world; sweet potatoes were chosen as they are increasingly cultivated and consumed in Europe and represent an important source of both phenolics and β-carotene [4,7].

## 2. Materials and Methods

### 2.1. Raw Vegetables

Fresh white cauliflowers (*Brassica oleacera* L.), carrots (*Daucus carota* L.) and orange-fleshed sweet potatoes (*Ipomoea batatas* L.) were purchased at a local supermarket, and each product came from the same producer. Vegetables were stored for a maximum of 24 h at +4 °C before the cooking trials. Cauliflowers were washed and drained, and florets with similar dimensions were separated from the bunch. Carrots were washed and peeled, the top and bottom ends were discarded and the root was cut in round slices 4 mm thick. Sweet potatoes were washed and peeled, then cut in slices 15 mm thick, from which cubes (15 × 15 × 15 mm) were obtained by means of a sharp grid. Products were divided into 300 g aliquots and submitted to the different cooking trials.

### 2.2. Cooking Conditions

Vegetables were cooked by three different methods, i.e., boiling, steaming and microwave cooking, for increasing times in order to reach various cooking levels, from insufficiently cooked to overcooked. The cooking times were established by preliminary trials and defined on the basis of the product texture, according to Italian eating habits.

Boiling: 300 g raw product were added to 2 L boiling unsalted tap water and removed from the water after the established times (5, 10, 15, 20 and 30 min for cauliflower and carrots; 4, 8, 12 and 16 min for sweet potatoes).

Steaming: the electric steamer (Daily Collection steamer, 9 L, 900 W, Philips) was preheated until the full development of vapor, then 300 g raw product were put in the basket, the lid was closed and the basket was removed after the established times (7.5, 15, 22.5 and 30 min for cauliflower and carrots; 4, 8, 12 and 16 min for sweet potatoes).

Microwaving: a microwave oven (Jet Chef Premium JT 479 WH, Whirlpool Corporation, VA, Italy) was used. Following the manufacturer’s instructions, 100 mL tap water were put on the bottom of the special vegetable cooking vessel, the raw product (300 g) was put on the perforated tray, the vessel was closed and put in the oven. Microwave cooking was performed at 500 W power, for the established times (5, 10, 15 and 20 min for cauliflower and carrots; 6, 8, 10, 15 and 20 min for sweet potatoes).

At the end of each cooking test, the product was removed from the cooking vessel, briefly drained on paper and put in aluminum food trays cooled by contact with ice for rapid cooling. When cooled, the products were immediately analyzed for their texture, color and aromatic profile, and the remaining samples were stored at −20 °C for subsequent analytical determinations.

For each vegetable, the different cooking trials were carried out in duplicate, on different days, to account for the variability in raw materials.

### 2.3. Texture

The texture of cooked vegetables was evaluated using a TA. HDplus Texture Analyser (Stable Micro System, UK) equipped with a 10-bladed Kramer Shear Cell. During the test, about 60 g of cooked vegetables (carrot slices, cauliflower florets and sweet potato cubes) were sheared, compressed and extruded through the bottom openings by the blades. The force required was continuously recorded. The following conditions were used: load cell, 250 kg; test speed, 2 mm/s; post-test speed, 10 mm/s; displacement, 60 mm. From the force–distance curve recorded, the maximum force (N, corresponding to the maximum force reached during the test) and energy (N*mm, corresponding to the area under the force–distance curve from the initial compression to the complete extrusion of the product through the bottom of the cell) were extrapolated. Each texture test was carried out in duplicate.

### 2.4. Analytical Determinations

When not specified, all reagents were analytical or HPLC grade.

Frozen samples were thawed overnight at +4 °C, then finely ground with a food mixer (Ariete mod. Speedy, Italy) before analysis.

Total solids were determined gravimetrically by drying the ground samples to a constant weight at 105 °C [17]. This determination was carried out in triplicate for each sample.

To determine the total phenolics (TP) and antioxidant activity (AA), a methanolic extract was prepared as follows: 5 g product was added to 20 mL pure methanol, the mixture was homogenized by Ultraturrax (T 25, IKA-Werke GmbH & Co.KG, Staufen, Germany) at 12,000 rpm for 1 min, then stirred for 60 min in the dark. The extract was separated by filtration (Whatman N. 4). Each extract was obtained in duplicate.

TP were determined by the Folin–Ciocalteau assay [18], as previously reported [19], and their concentration was expressed as gallic acid equivalents (GAE) by a calibration curve built with a pure standard of gallic acid (Sigma-Aldrich Italia). Determinations were carried out in duplicate.

AA was determined by the DPPH* (2,2-diphenyl-1-picrylhydrazyl) assay [20], modified as follows: 1 mL of suitably diluted extract was added to vials containing 5 mL of a 5 × 10^−4^ M methanolic solution of DPPH* (Sigma-Aldrich Italia). The vials were closed, mixed and left at 35 °C in a thermostatic bath for exactly 5 min, after which the absorbance at 515 nm (A) was read against a blank (1 mL of pure methanol added to 5 mL of DPPH* solution). The percentage of DPPH* inactivation was calculated as I% = (A sample – A blank)/A blank * 100, and data were converted into Trolox equivalents (TE) by comparison with a calibration curve built with Trolox (6-hydroxy-2,5,7,8-tetra-methyl-chroman- 2-carboxylic acid, Sigma-Aldrich Italia). Determinations were carried out in duplicate.

Ascorbic acid (ASC) in cauliflowers was determined by HPLC, following the method previously reported [21]. Briefly, 2.5 ground samples were extracted with 25 mL of a 0.003% metaphosphoric acid solution containing 50 mg/L Na_2_SO_3_. The suspension was homogenized by Ultraturrax for 1 min (12000 rpm), filtered (Whatman N. 4), diluted (1:3 *v/v*) with the mobile phase (0.002 M H_2_SO_4_), then microfiltered (0.45 micrometers acetate filters) and injected in the HPLC system, applying the conditions previously described [21]. A calibration curve was built with pure ascorbic acid. Each sample was analyzed in triplicate.

Carotenoids in the carrots and sweet potatoes were determined by HPLC. The extraction solvents were tetrahydrofuran added with 0.1% butyl-hydroxytoluene (BHT) for the carrots and acetone added with 0.1% BHT for the sweet potatoes. For both products, 2 g of the sample was mixed with 25 mL extracting solution, the suspension was homogenized for 1 min with Ultraturrax (12,000 rpm), then stirred for 30 min in the dark. Extracts were then filtered (Whatman N. 4), suitably diluted with the mobile phase, filtered at 0.22 micrometers and injected in the HPLC system. A C18 Vydac 201TP54 column (Alltech Italia) was used, eluted isocratically with methanol/tetrahydrofuran (95:5) at 1 mL/min, at room temperature. Peaks were detected at 454 nm and β-carotene and α-carotene were identified and quantified by a comparison with the calibration curves built with the pure standard compounds (Carotene mixed isomers from carrot, Sigma-Aldrich Italia). Each sample was analyzed in triplicate.

### 2.5. Electronic Nose

Analyses were performed with the portable PEN3 e-nose (Airsense Analytics, Schwerin, Germany). The system is composed of a sampling apparatus, a sensor chamber containing the sensor array and a pattern recognition software (Win Muster v.1.6) for the data recording and processing. The sensor array consists of 10 metal oxide semiconductor (MOS) sensors: W1C (aromatic), W5S (broad range), W3C (aromatic), W6S (hydrogen), W5C (aromatic–aliphatic), W1S (broad range), W1W (sulphur compounds), W2S (alcohols), W2W (sulphur compounds) and W3S (methane–aliphatic). The sensor response is expressed as resistivity (Ohm). Ten grams of the samples was placed in 100 mL Pyrex^®^ vials fitted with a pierceable silicon/teflon disk in the cap. After 1 h equilibration at room temperature, the measurement started. The headspace was pumped over the sensor surfaces for 60 s (injection time) at a flow rate of 400 mL min^−1^, and during this time the sensor signals were recorded. After each sample analysis, the system was purged for 180 s with filtered air in order to re-establish the instrument baseline before the injection of the subsequent sample. Each sample was evaluated in duplicate and the sensor drift was estimated by using a standard solution of 0.2% ethanol included in each measurement cycle.

### 2.6. Electronic Tongue

Analyses were performed with the Taste-Sensing System SA 402B (Intelligent Sensor Technology Co., Ltd., Atsugi, Japan). The detecting part of the system consists of potentiometric detecting sensors whose surfaces are combined with artificial lipid membranes having different response properties to the chemical compounds on the basis of their taste [22]. The detecting sensors used in this work were: CA0 for sourness, C00 for bitterness and aftertaste bitterness, AE1 for astringency and aftertaste astringency and AAE for umami. Ten grams of the sample was added to 100 mL of distilled water, stirred by Ultraturrax for 5 min at 15,000 rpm and hence centrifuged for 5 min at 9000 rpm. The aqueous extracts were submitted to the e-tongue procedure as reported by Buratti et al. [19]. Each sample was evaluated in duplicate and the sensor outputs were converted to “taste values” by using appropriate coefficients based on the Weber–Fechner law, which gives the intensity of sensation considering the sensor properties for tastes [22].

### 2.7. Electronic Eye

Color was measured as L* (lightness, black = 0, white = 100), a* (redness > 0, greenness < 0) and b* (yellowness > 0, blueness < 0) coordinates (CIE L*a*b* color space) using a tristimulus colorimeter Chroma Meter II (Minolta, Japan). The head of the colorimeter was directly applied to the surface of the vegetables (carrot slices, cauliflower florets and sweet potato cubes) and measurements were taken on 10 different pieces of the products to obtain the average L*, a* and b* values.

### 2.8. Statistics

Analytical data were processed by a Statgraphics Centurion (v. 18, Statistical Graphics Corp., Herndon, VA, USA). A one-way analysis of variance (ANOVA) was performed using the least significant difference (LSD) test to compare the sample means; differences were considered significant at *p* < 0.05.

E-senses data were transformed by column autoscaling and explored by a principal component analysis (PCA). PCA is an unsupervised exploratory procedure that allows the visualization of the relationships between the objects and variables in a reduced space, thanks to graphical outputs (i.e., score plot and loading plot) [23]. The PCA was performed by the Minitab 17 (v. 1.0, Minitab Inc., State Collage, PA, USA) software package.

## 3. Results and Discussion

### 3.1. Texture

**Figure 1:**
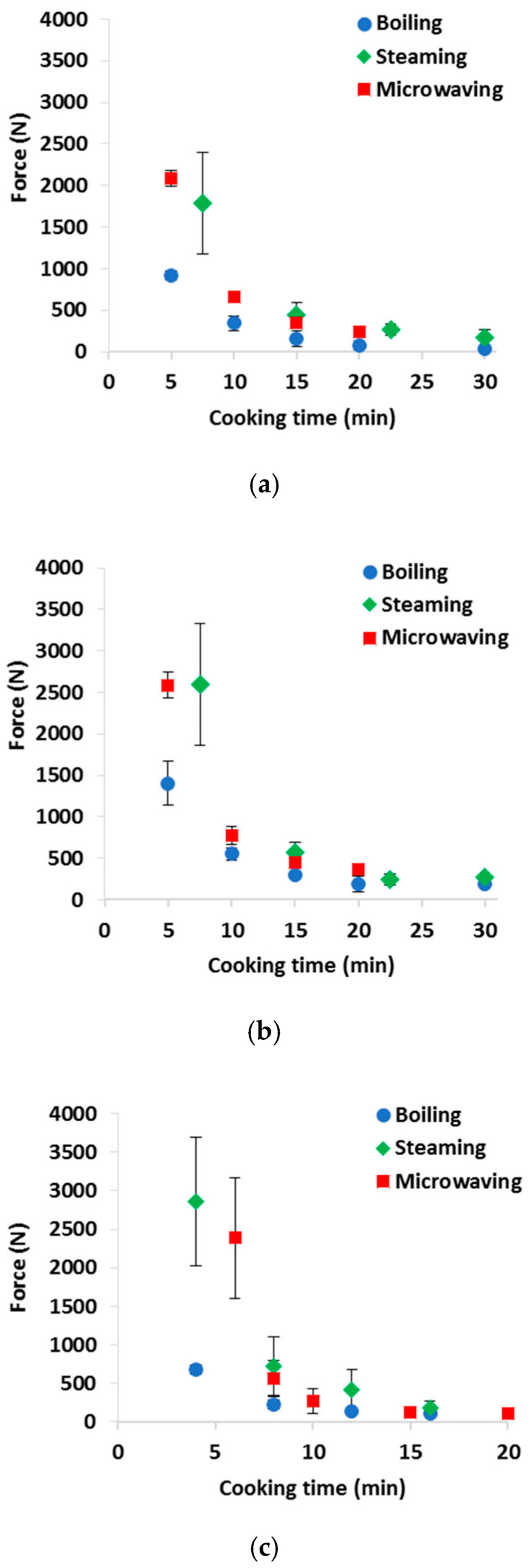
Texture evaluation of (**a**) cauliflowers, (**b**) carrots and (**c**) sweet potatoes cooked by different cooking systems and times. Data are averaged per trial and standard deviation bars are shown.

Figure 1 shows the texture data of the cauliflowers, carrots and sweet potatoes cooked by the different cooking systems and times. According to consumer preferences and vegetable type, different cooking times can be used. Bongoni et al. [11] reported that 65% of the consumers use mainly texture to decide the “doneness” of the vegetables: most consumers prefer a “hard texture” (e.g., 6–10 min boiling for broccoli) or “hard to soft texture” (e.g., 6–20 min boiling for carrots). In the present study, the cooking times were established by preliminary trials and defined on the basis of the product texture as determined by a shear stress extrusion test: a maximum force of approximately 500 N was considered as the optimal consistency according to Italian eating habits. As shown in Figure 1, for the first cooking time, all the vegetables were undercooked (force > 500 N). Though undercooked products could vary greatly in terms of hardness considering both the duplicated trials and cooking methods, the hardness of the samples became more similar at longer cooking times, regardless of the cooking method. For cauliflowers (Figure 1a) and carrots (Figure 1b), the target consistency (500 N) was reached after about 10 min of boiling or microwaving, whereas steaming required longer times; for sweet potatoes (Figure 1c), shorter cooking times were needed (4 to 8 min). Exceeding the optimal cooking times caused all the samples to become softer (lower force and energy values) and minor differences were evidenced according to the cooking method. For all products, boiling resulted in softer textures, even after the shortest cooking time.

### 3.2. Nutritional Properties

Table 1, Table 2 and Table 3 show the analytical data for cauliflowers, carrots and sweet potatoes cooked by boiling, steaming and microwaving at increasing times. Data are expressed on a dry weight basis, in order to account for the eventual dilution/concentration effects, and they refer to indexes typically related to the antioxidant power of each product. The results of the one-way ANOVA and multiple range test (Tukey test) are shown to evidence the effects of the different cooking times.

#### 3.2.1. Cauliflowers

The analytical data of the differently cooked cauliflowers are shown in Table 1. The solid content of raw cauliflowers varied from 7.6 to 9.0 g/100 g, depending on the lot. The cooking method influenced the moisture content: boiling produced a significant water uptake and the solid content progressively decreased to almost 60% of the initial value after 30 min. Steaming had a negligible effect on the moisture content and no specific trend could be observed during the cooking treatment. On the contrary, microwaving produced an increase in the solid content, due to water evaporation from the product despite the presence of water in the closed cooking vessel. Therefore, the microwaved cauliflowers showed a significantly higher solid content than the corresponding raw vegetables.

Concerning the antioxidant indexes, the ASC concentration detected in the raw cauliflowers was low, probably due to oxidative (mainly enzymatic) degradation during the extraction and analysis, as already reported [24,25]. Therefore, for each trial, the ASC was determined on the samples after 5 min boiling (blanching treatment), obtaining 29–43 mg/100 g, corresponding to 404–588 mg/100 g dry weight (dw). This initial content is in the range of the values reviewed by Podsedek [26]. Boiling had a detrimental effect on the ASC concentration, producing losses up to 50–60% (with respect to the 5 min boiling sample) after 30 min. The decrease was progressive and presumably due to both the thermal damage and dissolution into the boiling water. Steamed cauliflowers also showed losses in ASC, which were dependent on the cooking time, and a final loss of 40%. Microwaving resulted in minor changes in the ASC concentration and the final values were similar to the initial ones (blanched samples). Our findings only partly agree with previous research: although it is generally agreed that cooking produces significant losses in ASC and that boiling is more detrimental than steaming [27,28,29], microwave cooking (without water) has been reported to produce a high ASC degradation [28]. Bureau et al. [30] observed that the ASC retention was 77%, 94% and 88% for the boiled, steamed and microwaved cauliflowers, respectively, demonstrating that major losses in boiling were due to the dissolution into the boiling water.

The total phenolic content in the raw cauliflowers was in the range 350–500 mg GAE/100 g dw, corresponding to about 30–38 mg GAE/100 g fresh weight, in the range of the data in the literature [24,27,28,31]. The effect of cooking on TP varied according to the cooking method and time. The boiled cauliflower showed major losses in TP, which were related to the boiling time. Steaming and microwaving produced an increase in the TP content, related as well to the cooking time. These effects can be ascribed to the release of free phenolics derived from the hydrolysis of the high molecular weight compounds and to the disruption of the polyphenol–protein complexes, favored by heat treatment, and to the concurrent softening of the vegetable tissues which enhances both the extractability and solubility of the phenolic components. Similar conclusions have been reported for cauliflowers [24,27,32] and red cabbage [33].

The antioxidant activity, determined by the DPPH* radical scavenging assay, showed a particular trend in the cooked cauliflowers. The raw products had a low AA because of the low ASC and TP content. The values increased in the cooked products for all the cooking methods; in the boiled products, maximum values were detected after 10 min and then decreased, with a concurrent decrease in the ASC and TP content. Steamed and microwaved products showed an initial increase in AA, which maintained high values until the final cooking time, consistent with the TP and ASC concentrations. The low AA detected after short cooking times can be ascribed to some residual enzymatic oxidative activity that can interfere with the DPPH radical scavenging activity test.

Data about the AA of cooked brassica vegetables are controversial, as documented by review papers [10,26]. Some authors observed an increase in antioxidant activity after a cooking process [24,26,32], whereas others observed a decrease [24]. Volden et al. [27] reported high losses in the AA of boiled and steamed cauliflowers and showed that a major part of AA was recovered in the cooking medium (water).

#### 3.2.2. Carrots

The analytical data of the differently cooked carrots are shown in Table 2. The solid content of the carrots was highly influenced by the cooking system, as was observed for the cauliflowers. Boiling produced a progressive decrease (around a 40% decrease after 30 min), microwaving increased the solid content (about a 70% increase after 20 min) and steaming did not substantially change this parameter. The raw carrots had an initial content of 27–42 mg/100 g dw and 21–32 mg/100 dw for β-carotene and α-carotene (β-C and α-C), respectively, and these values increased during boiling and steaming, with similar behavior for the two carotenes. Maximum concentrations were detected after 20–30 min boiling, when three- to four-fold higher values of both β- and α-C were detected, and after 15–30 min steaming, with a two-fold increase for β-C and about a 1.5-fold increase for α-C. Microwave cooking had a different effect on the carotene concentrations, which increased slightly after 10–15 min, with final concentrations similar to the initial ones. The relatively low concentrations of carotenes detected in the raw carrots are due to the low extractability of these compounds, which are present as crystalline forms and can be associated with proteins and/or the membrane components; these forms exhibit low solubility and *in vitro* bioavailability and the relationship between the softening and bio-accessibility of carotenoids has been demonstrated [34]. Our data show that an enhancement of carotene accessibility was achieved by boiling and to a lesser extent by steaming. Microwave cooking had no positive effect on the carotene extractability, despite a softening of the vegetable tissue. Since microwaving is associated with water loss, we can assume that hydration of the tissues can help transform the insoluble forms of carotenes into more soluble ones. In the literature data, Mazzeo et al. [24] report that the boiling and steaming of frozen carrots produced a significant decrease in carotenes, while Miglio et al. [29] observed an increase in carotenes after boiling and a minor decrease after steaming. The TP in the raw carrots ranged from 108 to 140 mg/100 g dw, and their concentration differed according to how they were cooked. Boiling had a detrimental effect, producing losses of up to 20–30% after 30 min. The steamed carrots averaged 1.5-fold higher TP concentrations than the raw ones, with no clear trends related to the cooking time. Microwave cooking produced a significant increase in TP, whose concentrations after 15–20 min cooking were 1.4–1.8-times higher than the initial ones. An important loss of phenolic compounds due to boiling was previously reported [24,29], while other studies evidenced minor losses [32] or no losses [35]; when considering steaming, contradictory conclusions are found in the literature [24,29,32]; microwave cooking has been reported to increase the total phenolic content of carrots [32,35]. The AA of cooked carrots was undetectable in the raw products; we can assume that this result was due to the enzymatic oxidative activity that interfered with the antiradical scavenging activity, which is the basis of the DPPH test. Concerning the treated carrots, data were consistent with the TP content and not related to the carotene concentrations. Therefore, the maximum AA was detected in the steamed and microwaved carrots, whereas the minimum values were associated with long-boiled products.

#### 3.2.3. Sweet Potatoes

The data concerning sweet potatoes are shown in Table 3. The nutritional constituents evaluated in this vegetable were β-C, which represents the main carotenoid in orange-fleshed sweet potatoes [36], and TP, mainly represented by the phenolic acids in the orange-flesh roots [7,37].

The solid content of the sweet potatoes used in this research was 18.3 and 19.2 g/100 g for the two lots, respectively, which is lower than what is reported in the literature [7,38]. As already seen for the cauliflowers and carrots, boiling diminished the solid content of the product (approximately −20% after 16 min), steaming reduced this parameter to a lesser extent, while microwaving produced a significant increase, especially at cooking times of 15 and 20 min. The sweet potatoes had an initial β-C content of 33–39 mg/100 g dw (corresponding to 6.3–7.2 mg/100 g fresh weight), consistent with the data in the literature [36,39]. β-C concentrations increased in the sweet potatoes boiled for 4 min and remained almost stable for all the boiling times, with values 1.3- to 1.5-fold higher than the initial ones. Steaming produced no β-C losses in the first 12 min, but then a decrease (approximately 15–20%) was observed. The microwaved products showed a significant increase in the β-C levels at 6 and 8 min of the cooking time, and then the values decreased to final concentrations lower than the initial ones. As in the case of carrots, the increase in β-C in the heat-treated sweet potatoes can be explained by an enhancement of the extractability during the first period of cooking; carotenes then decreased due to the thermal damage and eventual leaching in the cooking medium. Little is known about the native form of carotenes in sweet potatoes, but these roots have a different composition and texture than carrots. In sweet potatoes, the release of carotene is faster and subsequent losses, due to thermal damage, occur. The literature data concerning the carotene stability in sweet potatoes lead to different conclusions, reporting a substantial stability regardless of the cooking method [40,41], but with minor losses [39] and major losses [42].

The TP in the raw products ranged from 187 to 210 mg/100 g dw, in agreement with the values reported by other authors [7,36]. In the boiled products, the TP content increased after 4 min, reaching a maximum concentration of 220 mg/100 g dw, then the concentrations stabilized around 200–210 mg/100 g dw. Steaming and microwaving resulted in a considerable increase in TP in the first 10 min, with the highest values detected in the steamed sweet potatoes (above 300 mg/100 g dw). For cooking times longer than 10 min, the steamed and microwaved products had similar and steady TP contents. An increase in TP as well as in individual phenolic acids in cooked sweet potatoes has been reported [7,37,42] and ascribed to various and concomitant factors: inactivation of polyphenoloxidases, hydrolysis of esterified products, release of bound phenolics from cell walls and insoluble fractions of the vegetable tissue, and the formation of Maillard reaction products that react with the Folin–Ciocalteau assay and thus contribute to overall quantification. The decrease observed in the sweet potatoes boiled for longer times can reasonably be ascribed to the dissolution of the soluble phenolic components (mainly phenolic acids) released by the above-mentioned modifications and favored by the water adsorption and softening of the cell walls. The AA of the sweet potatoes was low in the raw products (48–73 mg TE/100 g dw) and increased during cooking, for all the cooking methods. We can assume that raw roots exhibited a very low AA because of the presence of oxidative enzymatic activities, which are inactivated by cooking; accordingly, the low AA detected in the 4 min boiled samples could be due to an incomplete enzymatic inactivation. An increased AA was observed in the cooked samples, with the highest values in the steamed and microwaved products after 8 min. The boiled sweet potatoes showed a lower AA, with a declining trend at longer boiling times. Conflicting data about the effect of cooking on the AA are found in the literature, with some studies observing an increase [7,37] and others a decrease [36]. Values and trends observed in our tests are consistent with the phenolic content.

**Figure 2:**
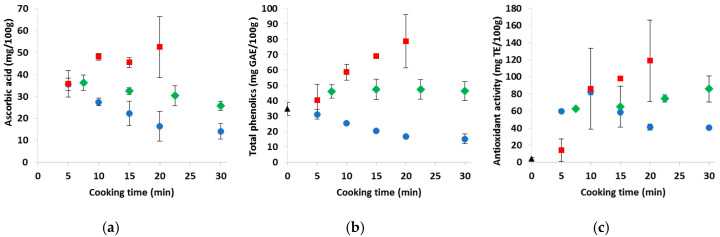

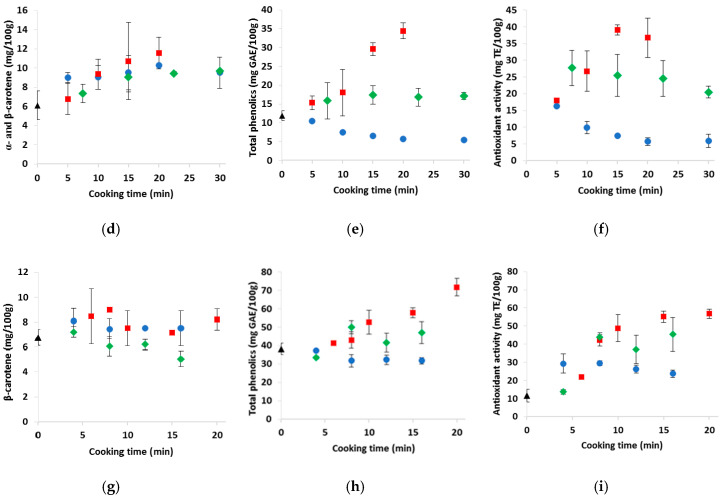
Nutritional properties of the (**a**–**c**) cauliflowers, (**d**–**f**) carrots and (**g**–**i**) sweet potatoes cooked by the different cooking systems and times. Data are averaged per trial and standard deviation bars are shown. GAE, Gallic acid equivalent; TE, Trolox equivalent; black triangle, raw samples; blue point, boiled samples; green diamond, steamed samples; red square, microwaved samples.

Figure 2 shows the nutritional indexes expressed per 100 g of the product as is, as the average of the two trials. The data allow us to make considerations about the nutritional value of the cooked products on an “as is” basis, i.e., as they are perceived by consumers. Concerning cauliflower (Figure 2, panels a, b and c), the evolution of ASC, TP and AA during cooking was clearly influenced by the cooking method and augmented by the dilution (boiling) and concentration (microwaving) effects. Therefore, the microwaved products showed the highest antioxidant indexes, while the boiled products showed the lowest. Considering an equivalent and optimal cooking degree (10 min for boiling and 15 min for steaming and microwave cooking), preference should be given to microwave cooking, followed by steaming; boiling represents the most detrimental method, especially for longer cooking times.

Concerning the carrots (Figure 2, panels d, e and f), the carotenoid concentration increased with the time and was not so affected by the cooking method. Differently, the TP and AA values were increased by microwave cooking and decreased by boiling, with more stable values with steaming. In this case too, when considering the optimal cooking time (10 min for boiling and 15 min for steaming and microwaving), the microwaved carrots should be preferred for their higher TP and AA values. As observed for cauliflower, prolonged boiling caused a significant depletion in TP and AA.

With regard to the sweet potatoes (Figure 2, panels g, h and i), the boiled and microwaved products had similar β-C contents, while the steamed samples had lower contents. The TP and AA values were related, in that they were increased by microwave cooking and diminished by boiling. Again, considering the optimal cooking time, which corresponded to about 4 min for boiling and 8 min for microwaving and steaming, the best results for these parameters were obtained with the microwaved and steamed products. As for the cauliflower and carrots, prolonged boiling was more detrimental than prolonged steaming or microwave cooking.

### 3.3. E-senses Results

E-senses (e-nose, e-tongue and e-eye) were applied in order to interpret the effects of boiling, steaming and microwaving on the sensory characteristics of the selected products; the data were combined with the texture parameters and elaborated by PCA into a quality evaluation system that encompasses the evaluation of smell, taste, color and texture, simulating a sensory analysis in a rapid and reliable way.

**Figure 3:**
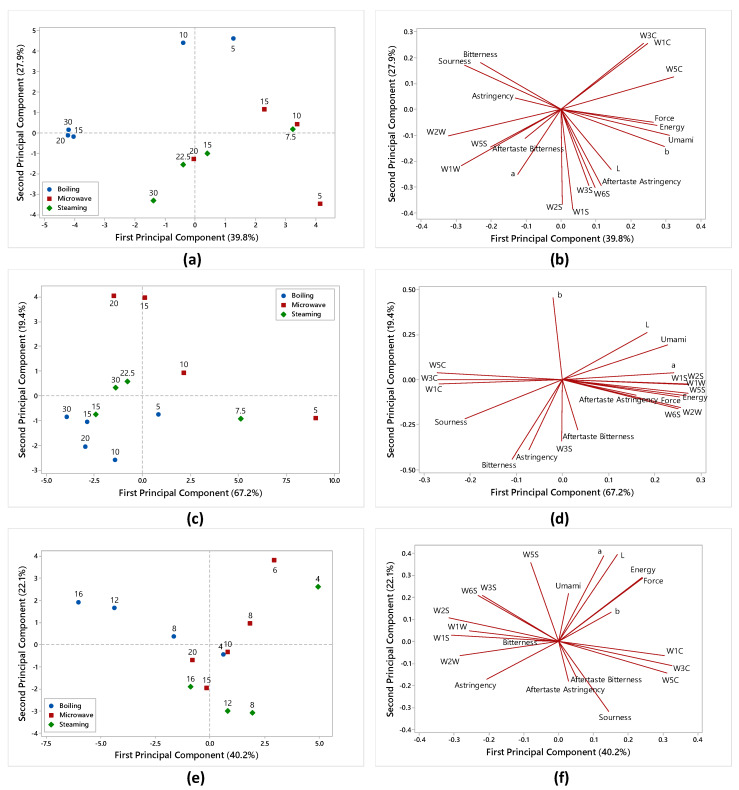
Principal component analysis (PCA) score plot and loading plot of e-senses and texture data collected on the (**a**,**b**) cauliflowers, (**c**,**d**) carrots and (**e**,**f**) sweet potatoes. In the score plots, the cooking time (min) is expressed by numbers.

Figure 3 shows the PCA score plots (a, c, e) and the loading plots (b, d, f) of the three selected vegetables.

The PCA score plot of the cauliflower (Figure 3a) indicates a clear evolution along the first principal component (PC1) (39.8% of total variance) for the boiled, steamed and microwaved samples according to the cooking time. In particular, the samples collected and analyzed at 5 min of boiling and up to 15 min for steaming and microwaving are located in the positive part of PC1 and are discriminated by the WC (W1C; W3C; W5C) e-nose sensors, specific for aliphatic and aromatic compounds, by texture parameters (force, energy) and by umami taste and astringency aftertaste as evaluated by the e-tongue (Figure 3b); as regards the color parameters, the samples cooked for shorter times are characterized by higher values for the lightness index (L*) and for the yellow index (b*).

For longer cooking times (i.e., after 10 min of boiling, 20 min of microwaving and 22.5 min of steaming), the samples moved to the left of the plot, indicating that the pattern of the cauliflowers changed during cooking, also in relation to the cooking methods. After 10 min of boiling, the samples were perceived as more bitter, sour and astringent, while over 15 min, they were characterized by the WW (W1W; W2W) e- nose sensors specific for sulphur organic compounds (Figure 3b). Changes in the sensory properties of the cauliflowers cooked by steaming and microwaving are quite similar and, after 20 min microwaving and 22.5 min of steaming, they appeared darker in color (higher a* values), discriminated by the WS e-nose sensors (W1S; W2S; W6S and W3S) of a broad range sensitivity and by the astringency aftertaste. These results are in agreement with the evolution of the nutritional parameters, since steamed and microwaved products showed a similar trend for the TP content and AA as well; moreover, the release of free phenolics and the disruption of the polyphenol–protein complexes favored by heat treatment can be related to the increase in bitterness and astringency during cooking.

The PCA score plot reported in Figure 3c shows the evolution of the carrots in the plane defined by the first two principal components (PC1 and PC2) (86,6% of the total variance) according to the cooking method and time. The samples cooked for shorter times (up to 10 min for microwaving, 7.5 min for steaming and 5 min for boiling) are positioned in the positive part of PC1 and are characterized by higher values of lightness (L*) and redness (a*); moreover, they are discriminated by the texture parameters, by umami taste and by the WW (W1W and W2W) and WS (W1S; W2S; W6S and W3S) e-nose sensors (Figure 3d). After 15 min of microwave cooking, the carrot samples, located in the upper part of the score plot, are mainly characterized by a change of color towards yellow as highlighted by the b* index value. The evolution in the plot of the samples cooked by steaming and boiling is similar and the samples collected after 10 min of boiling and 15 min of steaming, grouped in the negative part of PC1, are mainly characterized by the e-tongue variables such as sourness, bitterness and astringency, and by the WC e-nose sensors (W1C; W3C; W5C). These findings are in partial agreement with the nutritional parameters, since the boiled and steamed samples are characterized by a similar evolution of the β-carotene content, while during boiling there is a significant decrease in the TP content and AA. Moreover, for both cooking methods, the color parameters (L*, a*, b*) appear significantly decreased, thus indicating a gradual bleaching of the carrots during the treatments, as has been reported by Mazzeo et al. [24].

The PCA elaboration of the data collected on the sweet potatoes are reported in Figure 3e,f. As for the other two investigated products, the e-sense parameters evolve on PC1 (explained variance: 62.3%) according to the cooking time. In particular, the samples collected and analyzed at 4 min of boiling, up to 12 min for steaming and 10 min for microwaving are located in the positive part of PC1 and are discriminated by the WC (W1C; W3C; W5C) e-nose sensors (Figure 3f); the samples steamed and microwaved for the shortest cooking times (4 min for steaming and 6 min for microwaving) are located in the positive part of PC2 and are characterized by the texture parameters (higher values of energy and force) and by the color parameters (higher values of L*, a* and b*), indicating a more intense color of the samples. The samples steamed for 8 and 12 min are positioned in the negative part of PC2 and are mainly characterized by sourness, aftertaste astringency and aftertaste bitterness, as evaluated by the e-tongue. The samples boiled for 8, 12 and 16 min, discriminated in the negative part of PC1 by the WW and WS e-nose sensors, are mainly characterized by their aroma; this result is in accordance with Wang and Kays [43], who reported that sweet potatoes cooked by boiling and microwaving differ substantially both on a sensory and chemical level, and that the aroma intensity of microwaved sweet potatoes is lower than in boiled, resulting in the distinctly different aromas of the two products.

## 4. Conclusions

The work demonstrates that the nutritional properties and the sensory characteristics interpreted by the e-senses of the tested vegetables are strongly influenced by the cooking method and time. The nutritional parameters in the three products were affected by cooking in a different way. With regard to the cauliflowers, which are rich in hydrophilic antioxidants, boiling represented the most detrimental method, especially when longer cooking times were applied. The microwaved and steamed cauliflowers showed the highest antioxidant concentration and activity, even when overcooked. With regard to the carrots, boiling resulted in the highest release of carotenes but progressively reduced the TP concentration and AA. Therefore, considering both the hydrophilic and lipophilic antioxidants, 15 min steaming and microwave cooking produced the best results in terms of the antioxidant profile. Concerning the orange-fleshed sweet potatoes, which have a softer texture than carrots, the best results in terms of the nutritional quality were obtained by steaming and microwaving for intermediate cooking times, as these conditions maximized the β-C recovery and corresponded to the highest TP and AA values. For all products, the water content increased during boiling, decreased during microwaving and remained substantially unchanged during steaming; the concentration/dilution effect is significant when considering the amount of nutrients supplied by the equal amounts of the cooked products. The sensory parameters, interpreted by e-senses, showed a clear evolution according to the cooking method and time, and were often in agreement with the physico-chemical parameters. Changes in the sensory characteristics of the cauliflowers were similar for steaming and microwaving and, for longer cooking times, the samples appeared darker and discriminated by astringency aftertaste; the boiled samples were perceived as more bitter, sour and astringent and characterized by the e-nose sensors specific for sulphur organic compounds. The carrots cooked by steaming and boiling had a similar evolution pattern and were mainly characterized by sourness, bitterness and astringency. As for the sweet potatoes, a clear aroma evolution was evidenced especially for the boiled samples.

These results indicate the optimum cooking method to preserve and enhance the nutritional and sensory properties of the vegetables selected for the study. Furthermore, this study enlarges the knowledge of rapid instrumental methods, such as e-senses, which can be applied to study how cooking affects the sensory characteristics.

## Figures and Tables

**Table 1:** Nutritional properties of cauliflowers cooked by different cooking systems and times.

Cooking System and Time (min)	Solids (g/100 g)		Ascorbic Acid (mg/100 g dw)		Total Phenolics (mg GAE/100 g dw)		Antioxidant Activity (mg TE/100 g dw)	
	*Trial 1*	*Trial 2*	*Trial 1*	*Trial 2*	*Trial 1*	*Trial 2*	*Trial 1*	*Trial 2*
**Boiling**								
Raw (t_0_)	7.9 ± 0.1f	7.6 ± 0.1b	nd	nd	403 ± 18d	500 ± 15e	41 ± 17a	72 ± 22a
5	6.8 ± 0.1e	7.2 ± 0.1b	550 ± 6d	467 ± 9c	490 ± 27e	403 ± 13d	866 ± 16bc	855 ± 28c
10	6.2 ± 0.1d	6.8 ± 0.4b	461 ± 6c	388 ± 16b	420 ± 29d	369 ± 8c	1376 ± 140d	1185 ± 27d
15	5.9 ± 0.1c	6.7 ± 0.3b	315 ± 1b	241 ± 8a	339 ± 17c	316 ± 1a	1005 ± 165c	868 ± 30c
20	5.5 ± 0.1b	5.2 ± 0.8a	213 ± 1a	266 ± 45a	293 ± 21b	337 ± 10b	793 ± 40b	745 ± 43b
30	5.2 ± 0.1a	4.5 ± 0.3a	222 ± 3a	236 ± 17a	248 ± 7a	391 ± 14d	797 ± 29b	885 ± 11c
*p*-value	***	**	***	***	***	***	***	***
**Steaming**								
Raw (t_0_)	8.6 ± 0.1b	9.0 ± 0.7abc	nd	nd	348 ± 6a	394 ± 20a	52 ± 23a	35 ± 2a
Blanching	-	-	588 ± 45c	405 ± 7b	-	-	-	-
7.5	8.5 ± 0.1b	8.8 ± 0.1a	397 ± 11b	441 ± 47b	507 ± 5b	558 ± 7c	748 ± 24c	704 ± 25b
15	8.7 ± 0.1b	9.1 ± 0.2bc	386 ± 18b	345 ± 33a	492 ± 20b	573 ± 7cd	556 ± 15b	904 ± 22d
22.5	8.4 ± 0.1ab	8.8 ± 0.1ab	399 ± 2b	307 ± 11a	509 ± 8b	586 ± 8d	850 ± 34d	879 ± 108cd
30	8.2 ± 0.2a	9.3 ± 0.1c	334 ± 9a	261 ± 7a	621 ± 15c	452 ± 6b	1184 ± 35e	807 ± 50c
*p*-value	*	*	*	*	***	***	***	***
**Microwaving**								
Raw (t_0_)	8.5 ± 0.6a	8.0 ± 0.7a	nd	nd	390 ± 43a	413 ± 8a	94 ± 17a	104 ± 15a
Blanching	-	-	472 ± 32c	404 ± 33a	-	-	-	-
5	9.3 ± 0.1b	9.1 ± 0.1b	340 ± 20a	439 ± 12b	358 ± 6a	524 ± 7b	251 ± 34b	255 ± 15b
10	12 ± 0.1c	9.6 ± 0.1c	420 ± 6b	487 ± 4c	532 ± 18b	571 ± 12c	1021 ± 43d	547 ± 26c
15	12 ± 0.2d	11 ± 0.3e	367 ± 10ab	426 ± 9ab	574 ± 10c	625 ± 6d	800 ± 11c	905 ± 17e
20	15 ± 0.1e	10 ± 0.1d	425 ± 39b	411 ± 4a	616 ± 19d	640 ± 7e	1032 ± 4d	821 ± 20d
*p*-value	***	***	*	**	***	***	***	***

GAE, Gallic acid equivalent; TE, Trolox equivalent; nd, not detectable; -, blanching treatment not applied; t_0_, uncooked sample; *p*-value: *, *p* < 0.05; **, *p* < 0.01; ***, *p* < 0.001; within each cooking trial, values followed by different letters in the same column are significantly different (*p* < 0.05).

**Table 2:** Nutritional properties of carrots cooked by different cooking systems and times.

Cooking System and Time (min)	Solids (g/100 g)		β-Carotene (mg/100 g dw)		α-Carotene (mg/100 g dw)		Total Phenolics (mg GAE/100 g dw)		Antioxidant Activity (mg TE/100 g dw)	
	*Trial 1*	*Trial 2*	*Trial 1*	*Trial 2*	*Trial 1*	*Trial 2*	*Trial 1*	*Trial 2*	*Trial 1*	*Trial 2*
**Boiling**										
Raw (t_0_)	9.7 ± 0.1e	10.3 ± 0.6d	37 ± 2a	27 ± 6a	24 ± 6a	21 ± 9a	140 ± 30c	121 ± 11d	nd	nd
5	8.2 ± 0.1d	8.2 ± 0.1c	67 ± 12b	65 ± 3b	41 ± 7b	48 ± 3b	132 ± 10c	124 ± 4d	202 ± 9b	198 ± 4d
10	6.9 ± 0.1c	6.7 ± 0b	69 ± 2b	87 ± 4c	49 ± 0bc	60 ± 3c	109 ± 2b	108 ± 3c	126 ± 4a	166 ± 5c
15	5.9 ± 0.1b	6.5 ± 0.1b	81 ± 6bc	97 ± 2d	60 ± 6c	65 ± 5cd	108 ± 1ab	102 ± 5b	131 ± 6a	110 ± 8b
20	5.0 ± 0.2a	5.8 ± 0.2a	125 ± 8d	107 ± 2e	79 ± 7d	70 ± 1de	114 ± 3b	97 ± 4b	130 ± 1a	85 ± 20a
30	5.8 ± 0.1b	5.8 ± 0.7a	86 ± 2c	109 ± 6e	59 ± 0c	74 ± 1e	99 ± 7a	88 ± 3a	125 ± 5a	77 ± 12a
*p*-value	***	***	***	***	***	***	***	***	***	***
**Steaming**										
Raw (t_0_)	9.7 ± 0.1b	10.3 ± 0.6b	27 ± 2a	42 ± 6a	21 ± 6a	32 ± 9a	124 ± 30a	107 ± 11a	nd	nd
7.5	10.6 ± 0.1e	10.1 ± 0.1b	39 ± 13a	37 ± 2a	30 ± 11ab	29 ± 3a	202 ± 4d	102 ± 3a	297 ± 10b	183 ± 8a
15	10.2 ± 0.1d	8.0 ± 1.7a	57 ± 5b	60 ± 2b	43 ± 1b	39 ± 1b	187 ± 13c	196 ± 18c	292 ± 24b	264 ± 3d
22.5	9.4 ± 0.1a	9.2 ± 0.1ab	59 ± 1b	61 ± 1b	42 ± 0b	41 ± 1b	197 ± 9cd	163 ± 17b	302 ± 6b	225 ± 4c
30	10.0 ± 0.1c	9.3 ± 0.1ab	57 ± 1b	63 ± 0b	40 ± 2b	42 ± 1b	164 ± 6b	192 ± 7c	218 ± 3a	208 ± 2b
*p*-value	***	*	*	***	*	**	***	***	***	***
**Microwaving**										
Raw (t_0_)	9.7 ± 0.1a	10.6 ± 0.6a	37 ± 2	42 ± 6ab	24 ± 6a	32 ± 9ab	140 ± 30a	108 ± 11a	nd	nd
5	10.2 ± 0.1b	11.0 ± 0.1b	40 ± 5	38 ± 4a	24 ± 3a	26 ± 4a	163 ± 3b	128 ± 6b	177 ± 7a	164 ± 2a
10	12.4 ± 0.1c	10.6 ± 0.1a	44 ± 2	49 ± 3bc	32 ± 2b	38 ± 2b	181 ± 4c	128 ± 3b	275 ± 4c	182 ± 2b
15	14.4 ± 0.1d	15.3 ± 0.3c	33 ± 11	54 ± 1c	20 ± 5a	36 ± 0b	196 ± 9d	202 ± 10d	278 ± 4c	249 ± 8c
20	17 ± 0.3e	17.4 ± 0.3d	41 ± 2	40 ± 2a	25 ± 1a	29 ± 2a	211 ± 8e	189 ± 8c	241 ± 2b	187 ± 3b
*p*-value	***	***	ns	*	*	*	***	***	***	***

GAE, Gallic acid equivalent; TE, Trolox equivalent; nd, not detectable; t_0_, uncooked sample; *p*-value: ns, not significant; *, *p* < 0.05; **, *p* < 0.01; ***, *p* < 0.001; within each cooking system, values followed by different letters in the same column are significantly different (*p* < 0.05).

**Table 3:** Nutritional properties of sweet potatoes cooked by different cooking systems and times.

Cooking System and Time (min)	Solids (g/100 g)		β-caroTene (mg/100 g dw)		Total Phenolics (mg GAE/100 g dw)		Antioxidant Activity (mg TE/100 g dw)	
	*Trial 1*	*Trial 2*	*Trial 1*	*Trial 2*	*Trial 1*	*Trial 2*	*Trial 1*	*Trial 2*
**Boiling**								
Raw (t_0_)	19.2 ± 0.2d	18.3 ± 0.2d	33 ± 2a	39 ± 4	187 ± 4a	210 ± 9cd	48 ± 3a	73 ± 8a
4	16.2 ± 0.1c	17.2 ± 0.1c	46 ± 4b	51 ± 3	229 ± 9c	217 ± 8d	204 ± 4c	149 ± 13b
8	15.4 ± 0.1b	15.8 ± 0.2b	45 ± 8b	51 ± 5	222 ± 2bc	185 ± 8a	198 ± 2c	180 ± 8c
12	16.1 ± 0.1c	15.9 ± 0.1b	47 ± 4b	47 ± 2	212 ± 11b	192 ± 12ab	154 ± 6b	175 ± 6c
16	14.8 ± 0.1a	15.1 ± 0.2a	44 ± 1b	46 ± 4	223 ± 9bc	202 ± 8bc	150 ± 5b	167 ± 17c
*p* value	***	***	*	ns	***	***	***	***
**Steaming**								
Raw (t_0_)	19.2 ± 0.2c	18.3 ± 0.2d	33 ± 2ab	39 ± 4	187 ± 4a	210 ± 9b	48 ± 3a	73 ± 8a
4	17.9 ± 0.1b	17.9 ± 0.2c	42 ± 2c	38 ± 0	183 ± 12a	190 ± 5a	81 ± 17b	72 ± 1a
8	17.0 ± 0.2a	17.9 ± 0.1c	32 ± 5ab	37 ± 1	309 ± 7c	265 ± 9c	249 ± 6e	249 ± 16b
12	16.9 ± 0.1a	15.8 ± 0.1a	38 ± 0bc	37 ± 7	225 ± 10b	288 ± 7d	187 ± 7c	271 ± 12c
16	17.9 ± 0.2b	16.9 ± 0.1b	26 ± 4a	33 ± 2	240 ± 9b	302 ± 6e	217 ± 1d	308 ± 3d
*p* value	***	***	*	ns	***	***	***	***
**Microwaving**								
Raw (t_0_)	19.2 ± 0.2b	18.3 ± 0.2a	33 ± 2ab	39 ± 4ab	187 ± 4a	210 ± 9a	48 ± 3a	73 ± 8a
6	19.0 ± 0.1a	19.0 ± 0.3b	37 ± 2b	53 ± 1c	218 ± 3c	215 ± 8a	113 ± 6b	117 ± 8b
8	19.6 ± 0.1c	19.2 ± 0.2b	45 ± 1c	47 ± 1bc	204 ± 7b	241 ± 7c	227 ± 11c	208 ± 7d
10	19.9 ± 0.2d	21.0 ± 0.1c	33 ± 3ab	41 ± 5b	288 ± 9f	229 ± 9b	271 ± 6d	208 ± 10d
15	22.3 ± 0.1e	23.4 ± 0.1d	32 ± 4ab	31 ± 4a	250 ± 10d	255 ± 7d	257 ± 19d	226 ± 16e
20	25.0 ± 0.2f	28.8 ± 0.3e	26 ± 1a	31 ± 1a	273 ± 11e	261 ± 5d	234 ± 11c	191 ± 12c
*p* value	***	***	**	**	***	***	***	***

GAE, Gallic acid equivalent; TE, Trolox equivalent; t_0_, uncooked sample; *p*-value: ns, not significant; *, *p* < 0.05; **, *p* < 0.01; ***, *p* < 0.001; within each cooking system, values followed by different letters in the same column are significantly different (*p* < 0.05).

## References

[1] Kiran K.S., Padmaja G. (2003). Inactivation of trypsin inhibitors in sweet potato and taro tubers during processing. Plant Food Hum. Nutr..

[2] Gupta K., Wagle D.S. (1988). Nutritional and antinutritional factors of green leafy vegetables. J. Agr. Food Chem..

[3] Turkmen N., Sari F., Velioglu Y.S. (2005). The effect of cooking methods on total phenolics and antioxidant activity of selected green vegetables. Food Chem..

[4] Bengtsson A., Larsson Alminger M., Svanberg U. (2009). In vitro bioaccessibility of β-carotene from heat-processed orange-fleshed sweet potato. J. Agr. Food Chem..

[5] Rock C.L., Lovalvo J.L., Emenhiser C., Ruffin M.T., Flatt S.W., Schwartz S.J. (1998). Bioavailability of β-carotene is lower in raw than in processed carrots and spinach in women. J. Nutr..

[6] Guillén S., Mir-Bel J., Oria R., Salvador M.L. (2017). Influence of cooking conditions on organoleptic and health-related properties of artichokes, green beans, broccoli and carrots. Food Chem..

[7] Nicoletto C., Vianello F., Sambo P. (2018). Effect of different home-cooking methods on textural and nutritional properties of sweet potato genotypes grown in temperate climate conditions. J. Sci. Food Agric..

[8] Rennie C., Wise A. (2010). Preferences for steaming of vegetables. J. Hum. Nutr. Diet..

[9] Chuah A.M., Lee Y.C., Yamaguchi T., Takamura H., Yin L.J., Matoba T. (2008). Effect of cooking on the antioxidant properties of coloured peppers. Food Chem..

[10] Palermo M., Pellegrini N., Fogliano V. (2014). The effect of cooking on the phytochemical content of vegetables. J. Sci. Food Agric..

[11] Bongoni R., Verkerk R., Steenbekkers B., Dekker M., Stieger M. (2014). Evaluation of different cooking conditions on broccoli (Brassica oleracea var. italica) to improve the nutritional value and consumer acceptance. Plant Foods Hum. Nutr..

[12] Bongoni R., Stieger M., Dekker M., Steenbekkers B., Verkerk R. (2014). Sensory and health properties of steamed and boiled carrots (Daucus carota ssp. sativus). Int. J. Food Sci. Nutr..

[13] Bongoni R. (2014). Consumers and Vegetables: Effects of Domestic Processing on Sensory and Health Properties.

[14] Kala A., Prakash J. (2006). The comparative evaluation of the nutrient composition and sensory attributes of four vegetables cooked by different methods. Int. J. Food Sci. Technol..

[15] Sliwinska M., Wisniewska P., Dymerski T., Namiesnik J., Wardencki W. (2014). Food analysis using artificial senses. J. Agric. Food Chem..

[16] Ouyang Q., Zhao J., Chen Q. (2014). Instrumental intelligent test of food sensory quality as mimic of human panel test combining multiple cross-perception sensors and data fusion. Anal. Chim. Acta.

[17] AOAC (2002). Official Methods of Analysis.

[18] Singleton V.L., Rossi J.A. (1965). Colorimetry of total phenolics with phosphomolybdic–phosphotungstic acid reagents. Am. J. Enol. Viticult..

[19] Buratti S., Benedetti S., Giovanelli G. (2017). Application of electronic senses to characterize espresso coffees brewed with different thermal profiles. Eur. Food Res. Technol..

[20] Brand-Williams W., Cuvelier M.E., Berset C. (1995). Use of a free radical method to evaluate antioxidant activity. LWT-Food Sci. Technol..

[21] Giovanelli G., Limbo S., Buratti S. (2014). Effects of new packaging solutions on physico-chemical, nutritional and aromatic characteristics of red raspberries (Rubus idaeus L.) in postharvest storage. Postharvest Biol. Technol..

[22] Kobayashi Y., Habara M., Ikezazki H., Chen R., Naito Y., Toko K. (2010). Advanced taste sensors based on artificial lipids with global selectivity to basic taste qualities and high correlation to sensory scores. Sensors.

[23] Wold S., Esbensen K., Geladi P. (1987). Principle component analysis. Chemom. Intell. Lab. Syst..

[24] Mazzeo T., N’Dri D., Chiavaro E., Visconti A., Fogliano V., Pellegrini N. (2011). Effect of two cooking procedures on phytochemical compounds, total antioxidant capacity and color of selected frozen vegetables. Food Chem..

[25] Vallejo F., Tomas-Barberan F.A., Garcia-Viguera C. (2002). Glucosinolates and vitamin C content in edible parts of broccoli florets after domestic cooking. Eur. Food Res. Technol..

[26] Podsedek A. (2007). Natural antioxidants and antioxidant capacity of Brassica vegetables: A review. LWT—Food Sci. Technol..

[27] Volden J., Borge G.I.A., Hansen M., Wicklund T., Bengtsson G. (2009). Processing (blanching, boiling, steaming) effects on the content of glucosinolates and antioxidant-related parameters in cauliflower (Brassica oleracea L. ssp. botyris). LWT-Food Sci. Technol..

[28] Pellegrini N., Chiavaro E., Gardana C., Mazzeo T., Contino D., Gallo M., Riso P., Fogliano V., Porrini M. (2010). Effect of Different Cooking Methods on Color, Phytochemical Concentration, and Antioxidant Capacity fo Raw and Frozen Brassica Vegetables. J. Agric. Food Chem..

[29] Miglio C., Chiavaro E., Visconti A., Fogliano V., Pellegrini N. (2008). Effect of different cooking methods on nutritional and physicochemical characteristics of selected vegetables. J. Agric. Food Chem..

[30] Bureau S., Mouhoubi S., Touloumet L., Garcia C., Moreau F., Bedouet V., Renard C.M.G.C. (2015). Are folates, carotenoids and vitamin C affected by cooking? Four domestic procedures are compared on a large diversity of frozen vegetables. LWT—Food Sci. Technol..

[31] Proteggente A.R., Pannala A.S., Paganga G., Van Buren L., Wagner E., Wiseman S., van de put F., Dacombe C., Rice-Evans C.A. (2002). The antioxidant activity of regularly consumed fruit and vegetables reflects their phenolic and vitamin C composition. Free Radic. Res..

[32] Natella F., Belelli F., Ramberti A., Scaccini C. (2010). Microwave and traditional cooking methods: Effect of cooking on antioxidant capacity and phenolic compounds content on seven vegetables. J. Food Biochem..

[33] Podsedek A., Sosnowska D., Redzynia M., Koziolkiewicz M. (2008). Effect of domestic cooking on the red cabbage hydrophilic antioxidants. Int. J. Food Sci. Technol..

[34] Lemmens L., Van Buggenhout S., Oey I., Van Loey A., Hendrickx M. (2009). Towards a better understanding of the relationship between the β-carotene in vitro bio-accessibility and pectin structural changes: A case study on carrots. Food Res. Int..

[35] Faller A.L.K., Fialho E. (2009). The antioxidant capacity and polyphenol content of organic and conventional retail vegetables after domestic cooking. Food Res. Int..

[36] Wang S., Nie S., Zhu F. (2016). Chemical constituents and health effects of sweet potato. Food Res. Int..

[37] Chen Y., Xu Y., Cao Y., Fang K., Xia F., Jiang Q. (2017). Combined effect of microwave and steam cooking on phytochemical compounds and antioxidant activity of purple sweet potatoes. Food Sci. Technol. Res..

[38] Food Composition Database for Epidemiological Studies in Italy (Banca Dati di Composizione degli Alimenti per Studi Epidemiologici in Italia—BDA). European Institute of Oncology (2015). http://www.bda-ieo.it/wordpress/en/.

[39] Bengtsson A., Namutebi A., Larsson Alminger M., Svangberg U. (2008). Effects of various traditional processing methods on the all-trans-β-carotene content of orange-fleshed sweet potato. J. Food Compos. Anal..

[40] Donado-Pestana C.M., Mastrodi Salgado J., de Oliveira Rios A., dos Santos P.R., Jablonsky A. (2012). Stability of carotenoids, total phenolics and in vitro antioxidant capacity in the thermal processing of orange-fleshed sweet potato (Ipomea batatas Lam.) cultivars grown in Brazil. Plant Foods Hum. Nutr..

[41] Kuan L.Y., Thoo Y.Y., Siow L.F. (2016). Bioactive components, ABTS radical scavenging capacity and physical stability of orange, yellow and purple sweet potato (Ipomea batatas) powder processed by convection- or vacuum-drying methods. Int. J. Food Sci. Technol..

[42] Dincer C., Karaoglan M., Erden F., Tetik N., Topuz A., Ozdemir F. (2011). Effects of baking and boiling on the nutritional and antioxidant properties of sweet potato [Ipomoea batatas (L.) Lam.] cultivars. Plant Foods Hum. Nutr..

[43] Wang Y., Kays S.J. (2001). Effect of cooking method on the aroma constituents of sweet potatoes [Ipomoea batatas (L.) Lam.]. J. Food Qual..

